# EGFL7 reduces CNS inflammation in mouse

**DOI:** 10.1038/s41467-018-03186-z

**Published:** 2018-02-26

**Authors:** Catherine Larochelle, Timo Uphaus, Bieke Broux, Elizabeth Gowing, Magdalena Paterka, Laure Michel, Nevenka Dudvarski Stankovic, Frank Bicker, Florent Lemaître, Alexandre Prat, Mirko H. H. Schmidt, Frauke Zipp

**Affiliations:** 1 Department of Neurology, Focus Program Translational Neuroscience (FTN), Research Center Immunotherapy (FZI), Rhine Main Neuroscience Network (rmn2), University Medical Center of the Johannes Gutenberg University Mainz, Langenbeckstr. 1, 55131 Mainz, Germany; 2 Neuroimmunology Unit, Centre de Recherche du Centre Hospitalier de l’Université de Montréal (CRCHUM), Montréal, QC Canada; 3 Department of Neuroscience, Faculty of Medicine, Université de Montréal, 900 rue St-Denis, Montréal, H2X OA9 QC Canada; 4 Molecular Signal Transduction Laboratories, Institute of Microscopic Anatomy and Neurobiology, Focus Program Translational Neuroscience (FTN), Rhine Main Neuroscience Network (rmn2), University Medical Center of the Johannes Gutenberg University Mainz, Langenbeckstr. 1, 55131 Mainz, Germany; 5 German Cancer Consortium (DKTK), Im Neuenheimer Feld 280, 69120 Heidelberg, Germany; 6 German Cancer Research Center (DKFZ), Im Neuenheimer Feld 280, 69120 Heidelberg, Germany

## Abstract

Extracellular matrix (ECM) proteins secreted by blood-brain barrier (BBB) endothelial cells (ECs) are implicated in cell trafficking. We discovered that the expression of ECM epidermal growth factor-like protein 7 (EGFL7) is increased in the CNS vasculature of patients with multiple sclerosis (MS), and in mice with experimental autoimmune encephalomyelitis (EAE). Perivascular CD4 T lymphocytes colocalize with ECM-bound EGFL7 in MS lesions. Human and mouse activated T cells upregulate EGFL7 ligand αvβ3 integrin and can adhere to EGFL7 through integrin αvβ3. EGFL7-knockout (KO) mice show earlier onset of EAE and increased brain and spinal cord parenchymal infiltration of T lymphocytes. Importantly, EC-restricted EGFL7-KO is associated with a similar EAE worsening. Finally, treatment with recombinant EGFL7 improves EAE, reduces MCAM expression, and tightens the BBB in mouse. Our data demonstrate that EGFL7 can limit CNS immune infiltration and may represent a novel therapeutic avenue in MS.

## Abstract (web summary)

Endothelial cells release extracellular matrix components that regulate inflammation. Here the authors demonstrate that the extracellular matrix component epidermal growth factor-like protein 7 regulates inflammation in experimental autoimmune encephalomyelitis in the mouse.

## Introduction

The blood-brain barrier (BBB) maintains homeostasis of the central nervous system (CNS) by restricting cellular and molecular trafficking^1^. Expression of cell adhesion molecules (CAMs) by BBB endothelial cells (ECs) controls leukocyte migration into the CNS through interaction with their ligand expressed by peripheral blood leukocytes. In addition, astrocytes help to maintain BBB-ECs quiescence and integrity^2^. BBB disruption is a common and early feature in multiple sclerosis (MS)^3^, the most common chronic neuroinflammatory disorder, facilitating immune cell recruitment to the CNS^2,4,5^. Accordingly, natalizumab, a monoclonal antibody preventing interaction of α4 integrin-bearing leukocytes with vascular cell adhesion molecule (VCAM)-expressing BBB-ECs, substantially reduces accumulation of lesions and clinical relapses in MS^6^. However, since α4 integrin is expressed by most immune cells, its neutralization also affects the physiologic immune cell surveillance and compromises the protection against opportunistic CNS viral infections^7^. Identification of different therapeutic targets to modulate leukocyte-BBB interaction is therefore of growing interest in neuroinflammatory disorders such as MS.

Epidermal growth factor-like protein 7 (EGFL7), a protein secreted by ECs, is attached to the extracellular matrix (ECM) and interacts with integrin αvβ3 expressed by ECs to promote angiogenesis^8^. Furthermore, EGFL7 is upregulated under pathological conditions such as hypoxia^9^. Most importantly, EGFL7 helps ECs to survive under multiple stress conditions including low oxygen or low nutrients^10^. Although effects of EGFL7 on proliferation and activation of immune cells have not been reported so far, EGFL7 expression was associated with a reduced immune infiltration in breast cancer^11^. In line with this, EGFL7 was recently implicated in the regulation of peripheral EC activation through repression of the NF-κB pathway^12^.

The role of EGFL7 in neuroinflammation has not been studied, but as EGFL7 can influence EC survival and activation, we hypothesized that it could play a significant role in the animal model of MS, namely experimental autoimmune encephalomyelitis (EAE). Here, investigating EGFL7 in EAE, we demonstrate a beneficial role of EGFL7 in this neuroinflammatory disease model, reducing CNS immune cell infiltration as a consequence of BBB preservation and a reduction of CAM expression.

## Results

### Expression of EGFL7 in human BBB endothelial cells

**Fig. 1:**
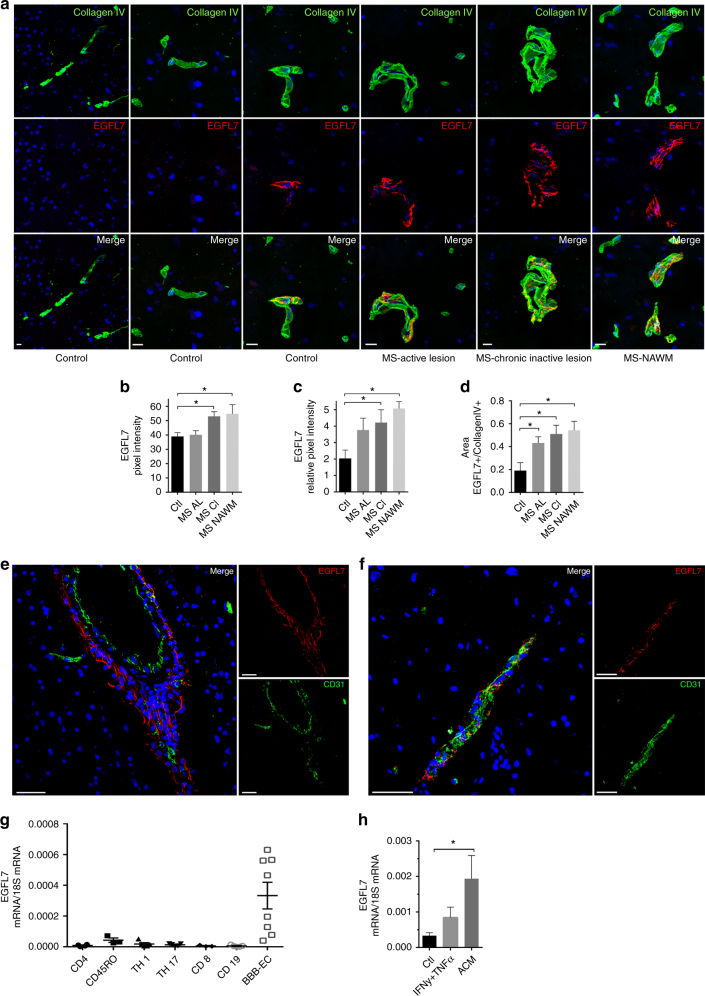
Expression of EGFL7 by human blood-brain barrier endothelial cells is altered in the CNS of MS subjects. **a** Expression of EGFL7 (red) by brain vessels (collagen IV, green) in human healthy control (Ctl, temporal), MS active lesion (AL, temporal), MS chronic inactive lesion (CI, temporal) and MS normal-appearing white matter (NAWM, frontal). Nuclei (DAPI) = blue. Representative of *n* = 5 MS patients and 5 Ctl. Scale bar = 10 μm. *n* ≥ 5 sections/subject were analyzed. **b**–**d** Semi-quantitative analysis of EGFL7 expression by BBB-ECs as presented in (**a**) according to (**b**) mean pixel intensity, (**c**) relative pixel intensity calculated as mean pixel intensity multiplied by area, and (**d**) area of positive signal for EGFL7 normalized to total vessel area (positive for collagen IV). **e**, **f** Expression of EGFL7 (red) in (**e**) areas of perivascular infiltration or (**f**) compared to healthy control tissue. CD31 expression (green), nuclei (blue). Scale bar = 50 μm. **g** Relative expression of EGFL7 mRNA by ex vivo human leukocytes sorted by CD4, CD8, CD14 or CD19 population, and in human BBB-ECs in primary culture. 1 dot = 1 donor. **h** Relative expression of EGFL7 mRNA by human BBB-ECs in primary culture in resting (Ctl), inflamed (TNF + IFN-γ 100 U/ml) or astrocyte-conditioned medium (ACM) conditions. *n* ≥ 8 different preps per condition. Statistical analysis performed by one-way ANOVA followed by Tukey’s multiple comparison test. *p < 0.05. All data presented as mean ± standard error of the mean (SEM)

EGFL7 is known to be expressed by different types of ECs. We here show that EGFL7 is also present at the adult human BBB in non-inflammatory control brain tissue (Fig. 1a and Supplementary Fig. 1a), and that expression of EGFL7 colocalized with the ECM protein collagen IV. Most interestingly, when compared to control human brain tissue, we observed a higher expression of EGFL7 in brain tissue from MS patients. Notably, based on a semi-quantitative analysis, EGFL7 expression in the ECM was significantly elevated in active lesions, but more so in chronic inactive lesions and normal-appearing white matter (NAWM) (Fig. 1b–d). Furthermore, the EGFL7 expression in MS tissue was found to follow a more scattered pattern (Fig. 1e, f). We also confirmed the expression of *egfl7* mRNA by human BBB-ECs in primary culture, but found no significant expression by human CD4, CD8, CD14 or CD19 leukocytes (Fig. 1g). To determine if pro-inflammatory or anti-inflammatory conditions can influence expression of EGFL7, we cultured BBB-ECs with medium (resting condition), IFN-γ/TNF (inflamed condition) or astrocyte-conditioned medium (ACM condition). We demonstrated an increase in EGFL7 expression as assessed by qRT-PCR after culture with ACM, which mimics the environment of a healthy BBB (Fig. 1h). These data support an increased expression of EGFL7 in MS CNS tissue, highest in NAWM and in EC following exposure to ACM, which might reflect a compensatory mechanism to overcome BBB disruption in neuroinflammation.

### T Lymphocytes express αvβ3 integrin and adhere to EGFL7

**Fig. 2:**
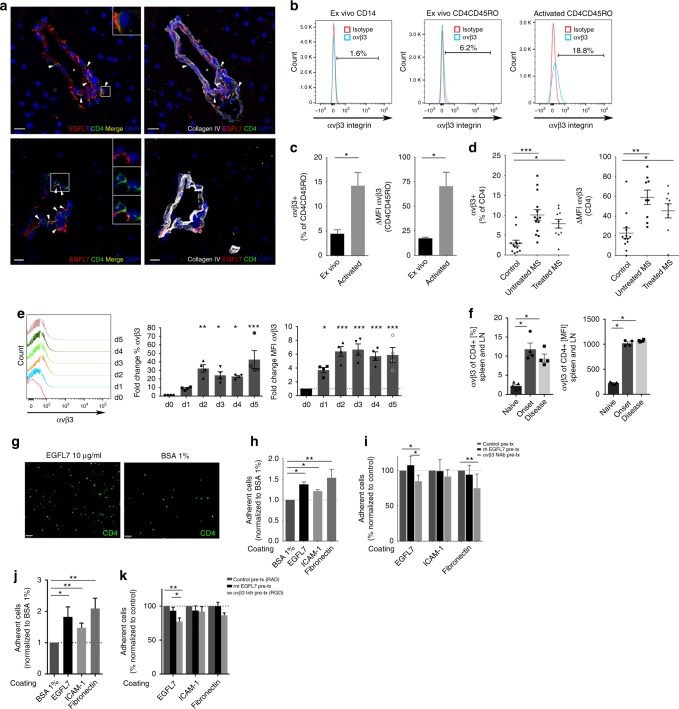
T lymphocytes express ligand αvβ3 integrin and adhere to EGFL7. **a** Colocalization of CD4+ cells (green) and EGFL7 (red) in perivascular ECM area in MS brain lesions (upper panel = frontal, lower panel = temporal). Collagen IV = white. Nuclei (DAPI) = blue. Representative of *n* = 4 MS donors, ≥ 3 lesions/donor. **b** Expression of αvβ3 integrin on human CD14 monocytes ex vivo (left), memory CD4 T lymphocytes ex vivo (center), and in vitro activated memory CD4 T lymphocytes (right), as assessed by flow cytometry. Representative of *n* = 6 donors. **c** Expression of αvβ3 integrin on human CD4 T lymphocytes ex vivo and upon activation. Percentage and mean fluorescence intensity (MFI). *n* = 6 donors. **d** Expression of αvβ3 integrin on human CD4 T lymphocytes ex vivo from healthy controls, untreated MS or treated MS. Flow cytometry analysis. 1 dot = 1 donor. **e**, **f** Expression of αvβ3 integrin by murine CD4 T lymphocytes (**e**) ex vivo and on day 1–5 in vitro upon activation, and (**f**) in naïve and MOG_35–55_-immunized (EAE) mice at different time points of EAE. *n* = 4–5 mice/condition, 1 dot = 1 mouse. **g**–**k** Adhesion assay. **g** Representative microscopy images of CFSE-labeled activated human CD4 T lymphocytes adhering to rhEGFL7 or BSA 1% after 24 h. Scale bar = 100 μm. Amount of human (**h**) and murine (**j**) activated CFSE-labeled CD4 T cells adhering to coated rh or rmEGFL7, ICAM-1 or fibronectin, normalized to BSA 1%, according to fluorescence intensity as assessed by plate-reader. *n* = 6 human donors and *n* = 8 mice. Amount of human (**i**) and murine (**k**) cells adhering to coated EGFL7, ICAM-1 or fibronectin, following pre-treatment with rhEGFL7 (**i**) or rmEGFL7 (**k**); anti-αvβ3 integrin neutralizing antibody (**i**) or RGD-peptide (**k**); or relevant control (rat serum, isotype or RAD-peptide). *n* = 7 human donors and *n* = 8 mice. Statistical analysis performed by Wilcoxon matched-pairs signed rank test (**c**) and one-way ANOVA followed by Tukey’s multiple comparison test (**d**–**k**). *p < 0.05, **p < 0.01, ***p < 0.001. All data presented as mean ± SEM

In contrast to BBB-ECs, T lymphocytes did not express relevant amounts of EGFL7 (Fig. 1g). The ligand of EGFL7, αvβ3 integrin, is not expressed by naïve T lymphocytes but can be induced by activation on mouse T lymphocytes^13,14^. We observed CD4+ cells in contact with EGFL7 strands in the ECM in MS brain lesions (Fig. 2a), suggesting that CD4 T lymphocytes could interact with EGFL7. We indeed show that a small percentage of ex vivo human CD4 memory T lymphocytes express αvβ3 integrin; this expression increased significantly after activation (Fig. 2b, c). Most importantly, αvβ3 integrin is upregulated on ex vivo CD4 T lymphocytes from untreated relapsing-remitting (RR) MS subjects and to a lesser extent from treated RRMS subjects, compared to healthy controls (Fig. 2d). We further observe a similar upregulation of αvβ3 integrin on the surface of murine CD4 T lymphocytes in EAE and upon activation (Fig. 2e, f). To address whether biologically significant interactions can take place between T lymphocytes and EGFL7, we performed an adhesion assay and found that adhesion of human CD4 T lymphocytes to coated recombinant human (rh)EGFL7 was significantly higher than to BSA 1%, but comparable to adhesion to coated ICAM-1 or fibronectin (Fig. 2g, h). Moreover, pre-treatment with rhEGFL7 did not significantly impact adhesion to coated EGFL7, ICAM-1 or fibronectin. This suggests that sustained/repeated interactions with immobilized EGFL7 are necessary for significant binding of CD4 T lymphocytes. Neutralizing αvβ3 integrin did significantly reduce adhesion to coated EGFL7, and as expected to fibronectin (Fig. 2i). However, exposure to plate-bound rhEGFL7 did not alter proliferation, cytokine production, or expression of CAMs or chemokine receptors in human (Supplementary Fig. 1b, c). Similar results were observed in murine CD4 T lymphocytes, as confirmed by αvβ3 integrin-mediated adhesion to coated recombinant murine (rm)EGFL7 (Fig. 2j, k), but again we found no significant impact of plate-bound rmEGFL7 on T lymphocyte polarization, activation or expression of CAMs (Supplementary Fig. 1d, e).

### EGFL7 expression is increased in the CNS of EAE mice

**Fig. 3:**
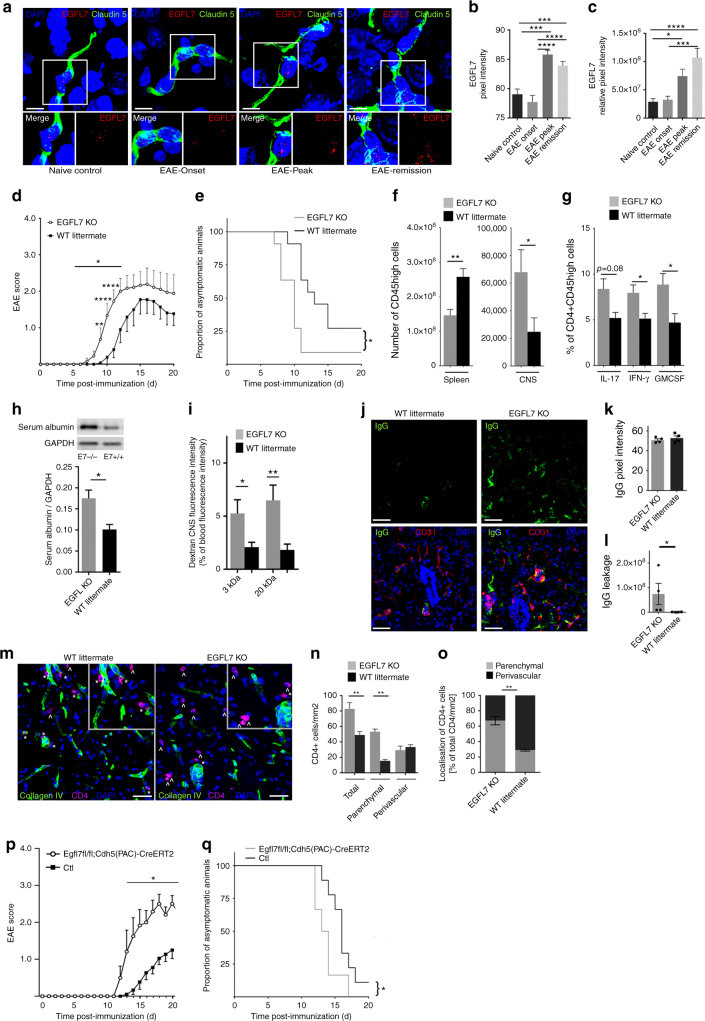
EGFL7-KO is associated with an earlier onset of EAE and an increased CNS immune cell infiltration. **a** EGFL7 expression (red) using FISH and immunofluorescence (claudin 5, green) in cerebellum, brainstem, and spinal cord of WT C57Bl/6 naïve control versus MOG_35–55_-immunized mice at EAE onset (d10), peak (d14) and remission (d25). Images (brainstem) representative of *n* ≥ 10 sections/mouse, 5 mice/time point. **b**, **c** Semi-quantitative analysis of EGFL7 expression according to (**b**) mean pixel intensity and (**c**) relative pixel intensity (mean pixel intensity × area). **d** EAE course and **e** proportion of asymptomatic mice in MOG_35–55_-immunized EGFL7*-*KO versus WT; *n* = 12 mice/group, two pooled independent experiments. **f** Numbers of CD45^High^ immune cells in spleen and CNS (brain and spinal cord) and **g** cytokine expression by CNS-infiltrating CD4 T lymphocytes. *n* = 5–6 mice/group, EAE d11. **h**, **i** BBB integrity in brain and spinal cord of EGFL7-KO versus WT: **h** Western Blot for serum albumin *n* = 5 mice/group, EAE d11; **i** in vivo permeability assay with fluorescently-labeled dextrans. *n* = 8 mice/group, EAE d13. **j**–**l** IgG leakage assessed by (**j**) immunofluorescence on spinal cord sections from representative EGFL7-KO or WT mice. **k**, **l** Semi-quantitative analysis of IgG signal (green) in CNS (cerebellum, brainstem, spinal cord) according to (**k**) mean and (**l**) relative pixel intensities. *n* ≥ 5 sections/mouse, 4 mice/group, EAE d10. **m**–**o** Localization of CD4+ cells in cerebellum, brainstem, and spinal cord of EGFL7-KO versus WT, *n* = 5 mice/group, EAE d13. **m** Representative images (brainstem). CD4+ cells (purple) without contact to collagen IV (green) considered invading into CNS parenchyma (^); cells in the immediate vicinity of collagen IV considered perivascular (*). **n** Total number/mm^2^ and **o** proportion of CD4+ cells invading CNS with respect to their localization. **p** EAE course and **q** proportion of asymptomatic mice in MOG_35–55_-immunized *Egfl7fl/fl;Cdh5(PAC)-CreERT2* males versus controls; *n* = 6 and 9 mice/group, respectively, two pooled independent experiments. Scale bars = 10 µm (**a**), 50 μm (**j**, **m**). Statistical analysis: one-way ANOVA with Tukey’s test (**b**, **c**), two-way repeated measures ANOVA with multiple comparisons test (**d**, **p**), Mantel-Cox test (**e**, **q**), Mann-Whitney *U*-test (**b**, **c**, **f**–**o**). *p < 0.05, **p < 0.01, ***p < 0.001, ****p < 0.0001. Data presented as mean ± SEM

As EGFL7 expression by BBB-ECs is altered in the brain of MS subjects, and its ligand αvβ3 is expressed by CD4 T lymphocytes, the next step was to determine if EGFL7 plays a role in the recruitment of immune cells across the BBB in central neuroinflammation in vivo in mice. First, we confirmed that EGFL7 is expressed by murine BBB-ECs using CNS material (cerebellum, brainstem, spinal cord) from non-immunized controls and MOG_35–55_-induced EAE mice. We found that on day 10 after EAE induction (day of onset), expression of EGFL7 was slightly decreased (intensity) or comparable (relative intensity) to expression in naïve control, however on day 14 (peak of disease) and most strikingly on day 25 (remission/chronic), similar to our observations in MS brain tissue, we observe that EGFL7 expression is increased in CNS vasculature of EAE mice (Fig. 3a–c).

### EGFL7*-*KO is associated with earlier onset of EAE pathology

To demonstrate that EGFL7 plays a role in neuroinflammation in vivo, we used EGFL7-knockout (KO) mice (Supplementary Fig. 1f)^15^. MOG_35–55_ immunization of EGFL7-KO mice resulted in a consistently and significantly earlier onset of EAE compared to littermates (Fig. 3d, e and Supplementary Table 1). As expected in light of the clinical course, on day 11 post-immunization the absolute number of peripheral immune cells infiltrating the CNS (whole brain and spinal cord) was significantly higher in EGFL7*-*KO compared to littermates, while the opposite was observed in the spleen (Fig. 3f). Among CD45^High^ cells, the proportion of CD4+ cells was significantly higher exclusively in the CNS of EGFL7-KO compared to littermates (CNS 18.2 ± 3.4% vs 5.7 ± 0.7%, *p* < 0.05), with no significant differences found in the proportion of CD11b (CNS 61.4 ± 4.6% vs 71.1 ± 3.8%), CD45R (CNS 12.7 ± 1.6% vs 12.6 ± 1.8%) or CD8 (CNS 2.6 ± 0.3% vs 1.8 ± 0.4%) subsets in the CNS or in the peripheral compartment. Among CD45^High^CD4+ T lymphocytes invading the CNS parenchyma, but again not those in the spleen, the proportion of pro-inflammatory IL-17+, IFN-γ+, and TNF+ cells was higher in EGFL7-KO than in littermates (Fig. 3g). Moreover, EGFL7-KO was associated with an increased leakage of serum albumin and fluorochrome-labeled dextran (whole brain and spinal cord), as well as fibrinogen and IgG (cerebellum, brainstem, and spinal cord) through the CNS vasculature of EAE mice, suggesting a role of EGFL7 in stabilizing the BBB in neuroinflammatory conditions (Fig. 3h–l and Supplementary Fig. 1g–j). In addition, we found a significantly higher number and proportion of CD4+ cells invading the CNS (cerebellum, brainstem, and spinal cord) parenchyma in EGFL7-KO mice, while the majority of CD4+ cells were confined to the perivascular space in wild-type (WT) littermates, suggesting that interaction of CD4 T lymphocytes with EGFL7 tethers cells to the ECM and prevents further migration into the CNS parenchyma (Fig. 3m–o). To exclude an unanticipated impact of EGFL7-KO on CD4 T lymphocyte migratory capacity, which could explain the earlier EAE onset in EGFL7-KO mice, we evaluated active EAE in *Rag2*^*−/−*^*cgn*^*−/−*^ mice reconstituted with either CD4 T lymphocytes isolated from EGFL7-KO mice or CD4 T lymphocytes from WT littermates. We did not observe an earlier onset or more severe course of EAE in mice reconstituted with EGFL7*-*KO CD4 T lymphocytes versus WT CD4 T lymphocytes (Supplementary Fig. 1k, l).

### miR-126/miR-126*-KO is not associated with more severe EAE

The *egfl7* gene harbors the microRNAs miR-126 and miR-126*, encoded by its intron 7, and the KO of *egfl7* can result in altered miR-126 and miR-126* expression^16^. Since miR-126 and miR-126* are associated with CNS vasculature abnormalities and regulation of CAM expression^17^ in addition to downregulation of EGFL7^18^, we assessed the course of EAE in miR-126/miR-126*-KO. No significant difference was observed in either onset or severity of EAE between miR-126/miR-126*-KO and WT littermates (Supplementary Fig. 1m, n), and a trend towards a reduced EAE severity was present, suggesting that there was no major contribution of miR-126/miR-126*-KO to the EAE worsening observed in EGFL7-KO. miR-126/miR-126* is considered the main driver of previously published EGFL7-KO-associated CNS vasculature abnormalities^18^, which however did not seem to contribute to worsen EAE clinical course. Moreover, active EAE in *Rag2*^*−/−*^*cgn*^*−/−*^ mice reconstituted with CD4 T lymphocytes isolated from miR-126/miR-126*-KO mice versus CD4 T lymphocytes from WT or heterozygous (Het) littermates was not associated with an earlier onset (d14-18 versus d13-17 post-immunization respectively) or a more severe course of EAE. Taken together, our data suggest that EGFL7 directly plays a protective role in EAE.

### EGFL7-KO restricted to ECs is associated with EAE worsening

To confirm that BBB-derived EGFL7 plays a beneficial role in neuroinflammation in vivo, we took advantage of a new tamoxifen-inducible conditional KO of EGFL7 restricted to vascular ECs (*Egfl7fl/fl;Cdh5(PAC)-CreERT2*) that spares miR-126/miR-126*^19^. We confirmed that tamoxifen treatment on day −4 and −3 before immunization was sufficient to induce Cre expression (Supplementary Fig. 1o), and used only male mice to reduce the impact of tamoxifen on EAE course. Herein we show that EGFL7-KO on ECs is associated with a significantly earlier onset of EAE and more severe course compared to controls (Fig. 3p, q), similar to our data in constitutional EGFL7-KO. Our data therefore outlines a beneficial role of EGFL7 at the level of the endothelium in EAE, which is not dependent on miR-126/miR-126*-KO or developmental abnormalities.

### EGFL7 treatment reduces neuroinflammation in EAE

**Fig. 4:**
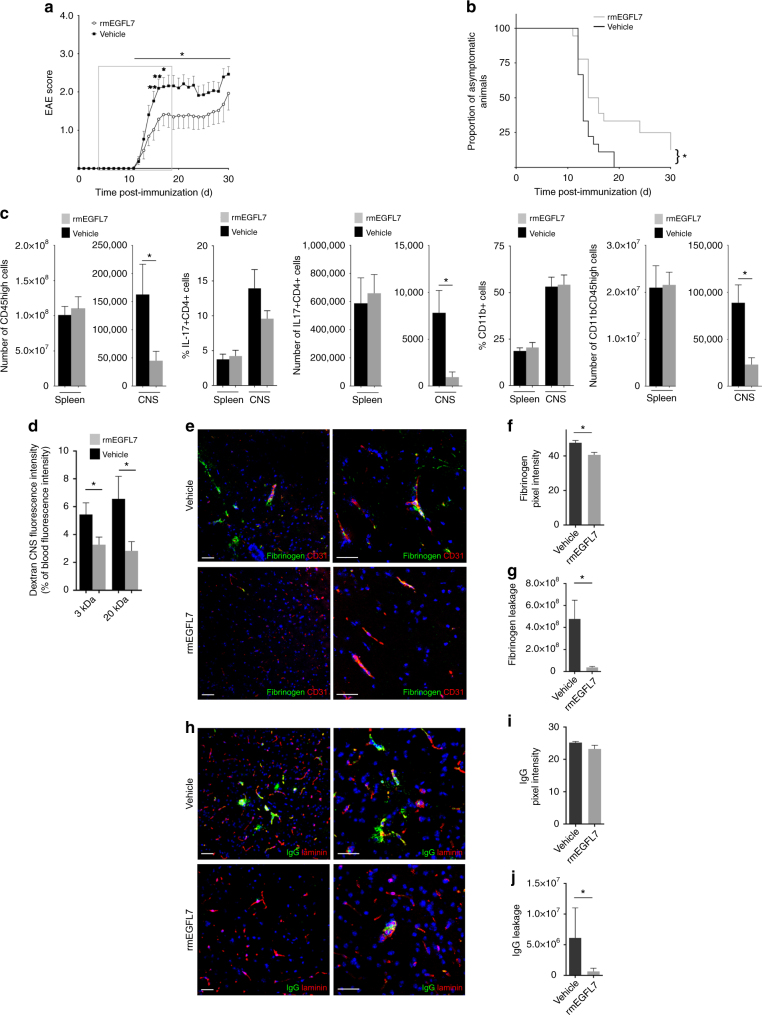
EGFL7 treatment ameliorates EAE disease course, preserves BBB and decreases immune cell infiltration into the CNS. **a**, **b** 8 to10-week-old wild-type (WT) C57Bl/6 female mice were immunized with MOG_35–55_ peptide. rmEGFL7 10 µg/ml or vehicle was administered intraperitoneally (i.p.) every other day from days 4–18 (grey box). *n* = 18 mice per group, from two pooled independent experiments. **a** EAE score. **b** EAE prevalence. **c** 2–3 days after EAE onset, representative mice were lethally anesthetized prior to perfusion (*n* = 5–6 control mice, from two pooled independent experiments) and leukocyte isolation before analysis of spleens and CNS (brain and spinal cord) by flow cytometry. **d** In vivo permeability assay in EAE following exposure to rmEGFL7 or vehicle, using intravenous injection of fluorescently-labeled dextrans (3, 20 kDa). Dextran extravasation to the CNS (brain and spinal cord) is expressed as a percentage of blood fluorescence intensity. *n* = 8 mice/group, d18. **e**–**j** BBB integrity as measured by fibrinogen (**e**–**g**) or IgG (**h**–**j**) leakage was assessed by immunofluorescence on frozen sections from representative EAE mice treated with rmEGFL7 (lower panels) or vehicle (upper panels). Scale bar = 50 μm, spinal cord. **f**, **i** Semi-quantitative analysis of fibrinogen or IgG signal (green) in the cerebellum, brainstem, and spinal cord according to mean pixel intensity and **g**, **j** leakage of fibrinogen or IgG according to relative pixel intensity (mean pixel intensity × area). *n* ≥ 5 sections per mouse, from *n* = 5 mice/group. Statistical analysis performed by two-way repeated measures ANOVA with multiple comparisons test (**a**), by Mantel-Cox test (**b**), and by Mann-Whitney *U*-test (**c**–**j**). *p < 0.05, **p < 0.01. All data presented as mean ± SEM

The lack of EGFL7 in KO mice was associated with a worse disease course and BBB breakdown during EAE, thus suggesting a beneficial role of EGFL7 in neuroinflammation. As proof of concept, and to exclude a potential contribution of EGFL7*-*KO-related developmental abnormalities or influence of tamoxifen, we induced EAE in C57Bl/6 mice and administered either rmEGFL7 or vehicle (intraperitoneally; i.p.). Treatment with rmEGFL7 resulted in a significantly delayed onset and milder disease course of EAE (Fig. 4a, b). Moreover, we found significantly lower numbers of CD45+ infiltrating leukocytes in the CNS (whole brain and spinal cord) of EGFL7-treated mice (Fig. 4c); in particular we observed a preferential reduction of IL-17-producing CD4 T lymphocytes, while only CD11b+ number but not proportion was reduced. As no difference was observed in the number and phenotype of peripheral immune cells following administration of rmEGFL7 (Fig. 4c), we assumed a protective effect on the BBB. We indeed could demonstrate a preservation of BBB functional integrity, underlined by a lower leakage of dextran (whole brain and spinal cord) as well as IgG and fibrinogen (cerebellum, brainstem and spinal cord) into the CNS parenchyma following rmEGFL7 exposure (Fig. 4d–j).

### EGFL7 treatment reduces MCAM expression in BBB-ECs

**Fig. 5:**
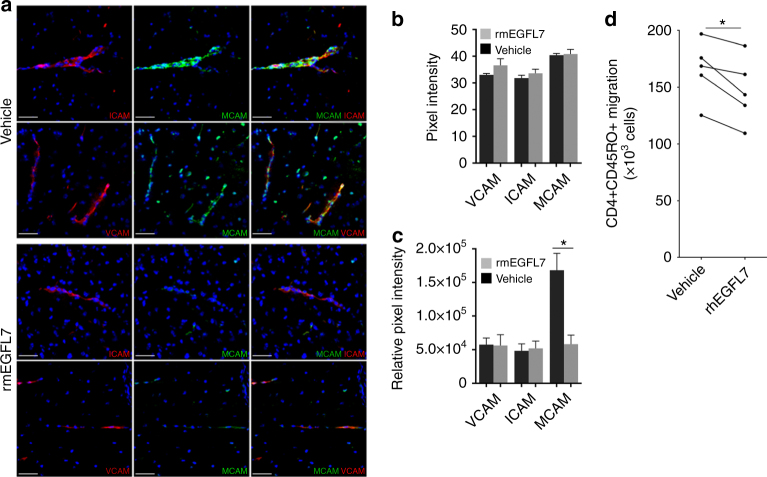
EGFL7 treatment decreases expression of MCAM by BBB-ECs. **a**–**c** Expression of cell adhesion molecules ICAM-1, MCAM, and VCAM-1 was assessed by immunofluorescence on frozen sections (cerebellum, brainstem, and spinal cord) from representative EAE mice treated with rmEGFL7 (lower panels, spinal cord) or vehicle (upper panels, spinal cord) as described. Left column: ICAM-1 or VCAM-1 (red), DAPI (blue). Middle column: MCAM (green), DAPI (blue). Right column: merge (yellow). Scale bar = 50 μm. *n* ≥ 5 sections/mouse, from *n* = 5 mice/group. **b**, **c** Pixel intensity and relative pixel intensity of ICAM-1, MCAM and VCAM-1 as calculated using ImageJ software. **d** Number of cells in bottom chamber after 16 h migration over inflamed human BBB-ECs (modified Boyden chamber assay), following 24 h exposure to rhEGFL7 10 μg/ml or vehicle. One million activated CD4CD45RO T lymphocytes were seeded. *n* = 5 donors, two different preps. Statistical analysis performed by Mann-Whitney *U*-test (**b**, **c**) and by Wilcoxon matched-pairs signed rank test (**d**). *p < 0.05. All data presented as mean ± SEM

As EAE mice treated with EGFL7 showed reduced CNS infiltration but no change of their peripheral immune cells profile, we next investigated if EGFL7 could interfere with lymphocyte transmigration by influencing expression of CAMs by BBB-ECs. A significant reduction of MCAM expression by BBB-ECs after EGFL7 treatment was noted (Fig. 5a–c), which could explain the reduced recruitment of CD4+ and most importantly Th17 lymphocytes across the BBB^20^. We also exposed inflamed human BBB-ECs in primary culture to rhEGFL7 or vehicle and measured expression of CAMs. We observed a significant reduction of MCAM mRNA, but not ICAM-1, ALCAM or VCAM-1, after treatment with rhEGFL7, to 0.63 ± 0.12 (SD) of levels in vehicle-treated BBB-ECs (*p* < 0.05). In line with this and with the EAE results, treatment with rhEGFL7 in an in vitro BBB model significantly reduced migration of activated human memory CD4 T lymphocytes over inflamed human BBB-ECs in primary culture (modified Boyden chamber assay), indicating a tighter BBB-ECs monolayer (Fig. 5d). Taken together, our findings indicate a beneficial role of EGFL7 in neuroinflammation in vitro and in vivo, which could prove of therapeutic interest in MS by uniquely promoting BBB function and reducing pro-inflammatory immune cell infiltration in the CNS.

## Discussion

The pathology of MS, the most frequent chronic CNS inflammation in North-Western countries, is initially driven by immune cell invasion of the CNS. Therefore, blocking transmigration of immune cells through the BBB, as for example with natalizumab, is very effective to achieve disease control in MS^7^. However, as with other highly effective disease-modifying therapies which influence a broad range of peripheral immune cells, potential devastating adverse events limit the use of this therapy as a first-line agent. This underlines the need to identify novel therapeutic strategies that display a more favorable risk-benefit ratio to restrict pathological CNS immune cell transmigration. Herein, we describe the role of the ECM protein EGFL7 in the regulation of pro-inflammatory lymphocyte trafficking through the BBB. We found an elevated expression of EGFL7 in the CNS of both human MS and its animal model EAE in subacute and chronic/remitting phase. In addition, expression in human ECs was upregulated upon treatment with astrocytic factors, known to promote BBB integrity. We demonstrated that T lymphocytes express EGFL7 ligand αvβ3 integrin and can adhere to EGFL7. Finally, while EGFL7-KO mice showed earlier onset of disease and increased BBB permeability, treatment with EGFL7 led to an amelioration of disease severity in EAE, as well as tightening of the BBB in vivo in mouse, thus representing a novel therapeutic principle in neuroinflammation.

Our data demonstrate that the CNS response to a chronic inflammatory attack, namely EAE in mice and MS in humans, leads to an upregulation of EGFL7 expression by BBB-ECs. Since EGFL7 is upregulated in angiogenesis, arterial injury, stroke^21^ or hypoxia^22^, our results in the MS and EAE CNS, where relative tissue hypoxia may constitute a pathologic mechanism^23^, is consistent with previous reports in non CNS endothelium. We also report that astrocyte-produced factors increase EGFL7 expression in human BBB-ECs, and we postulate that EGFL7 upregulation by BBB-ECs is induced as a compensatory mechanism to promote survival and recovery of BBB function in neuroinflammatory conditions and to limit the upregulation of CAMs, as indicated before in peripheral endothelium^10,11,24^. In this sense, the reduced CNS permeability seen in EGFL7-treated EAE mice and the decreased migration across a human in vitro BBB model, as well as downregulation in MCAM expression following exposure to recombinant EGFL7 both in human in vitro and in mice in vivo, are in support of the positive regulatory role of EGFL7 on BBB function. The strong induction of EGFL7 by anti-inflammatory astrocytic factors is reminiscent of previous reports of high expression of EGFL7 by ECs in tumors, considered a relatively immunosuppressive environment^25^.

EGFL7 is highly conserved in vertebrates, is important for vascular development and promotes cell proliferation and migration as well as repair, and regulates EC activation^9,10,26^. Hypoxia and metabolic stress enhance expression of EGFL7 in EC, which promotes endothelium survival and was reported to be mediated by HIF-1α^10,27,28^. In turn, EGFL7 is a Notch-antagonist^29^ and inhibits NF-kB activation^24^, considered a key negative regulator of BBB barrier function, which interferes with expression and stability of tight junction proteins^30^. This NF-kB-dependent inflammatory activation of ECs is regulated by EGFL7 via preservation of IkB-α and subsequent sequestration of NF-kB, which has been shown to downregulate ICAM-1 in human coronary ECs^24^. Our human in vitro and EAE in vivo data demonstrate that MCAM expression, rather than ICAM-1 or VCAM-1 as previously reported in peripheral endothelium^12,25^, is subjected to regulation by EGFL7 in BBB-ECs. This difference could depend on the type of cytokines present, which can impact both CAM and EGFL7 expression, on the type of endothelium and on the influence of astrocytic factors in vivo. In fact, as we and others have previously reported a crucial role of MCAM in encephalitogenic Th17 lymphocyte transmigration across the BBB in neuroinflammation^20,31^, the relatively specific modulation of MCAM expression on BBB-ECs by EGFL7 is in line with the observed preferential regulation of CNS infiltration by pathogenic Th17 cells in EGFL7-KO and in mice treated with rmEGFL7.

EAE has known limitations as a model for MS, but does recapitulate the main features of demyelination and neuroglial injury secondary to CNS infiltration by pro-inflammatory immune cells, while allowing in vivo studies. Before drawing conclusions from our findings in EGFL7-KO mice, however, we excluded an underlying role of the miRNA miR-126 and miR-126*, expressed in the non-coding intron 7 of the *EGFL7* gene, as the impact of this miR on the course of EAE has never been reported. Expression of miR-126 and miR-126* by ECs has been implicated in CNS vessel development^16^, and expression by leukocytes in autoimmune conditions^32–36^. However, our data revealed neither changes in onset nor severity of EAE between miR-126/miR-126*-KO and WT littermates that could account for the observed effects in EGFL7-KO. In fact, a trend towards a milder EAE was observed in miR-126/miR-126*-KO, the opposite of our observations in EGFL7-KO, therefore suggesting a direct beneficial effect of EGFL7 on EAE. This is further supported by the beneficial impact of rmEGFL7 treatment on neuroinflammation in vivo. Finally, our EAE data in a new inducible EGFL7-KO restricted to the endothelium that spares miR-126/miR-126* demonstrate that EAE worsening in the absence of EGFL7 is independent of its expression in non-endothelial cells, of developmental abnormalities, and of the expression of miR-126/miR-126*. Indeed, recent data supports the hypothesis that EGFL7 mediates its vascular effects beyond the functions of miR-126/miR-126*^37^.

EGFL7*-*KO did not cause similar worsening of active EAE course when restricted to CD4 T lymphocytes. Considering this and the fact that EGFL7*-*KO was not associated with altered number or proportion of pro-inflammatory leukocytes in the peripheral compartment in EAE, and that treatment with recombinant EGFL7 did not modify the phenotype of peripheral immune cells in vitro and in vivo or their adhesion to EGFL7, we concluded that the impact of EGFL7 on CNS immune cell infiltration was most probably at the level of the BBB-ECs and ECM. Our data on CAM expression and dextran/fibrinogen/IgG leakage support such a conclusion, which is further strengthened by the observed worsening of EAE in the EC-restricted EGFL7-KO. Moreover, our data show that CD4 T lymphocyte express the ligand of EGFL7 αvβ3, and more so in inflammatory conditions such as MS and EAE, in line with previous studies showing that αv integrin and β3 integrin are individually upregulated in MS^38^. CD4 T lymphocytes can bind to coated EGFL7 through αvβ3 integrin, and this interaction seems to tether cells to the ECM, as we observed CD4+ cells in contact with EGFL7 strands in the ECM in MS lesions, and as KO of EGFL7 was associated with a more efficient migration of CD4+ cells into the CNS parenchyma from the perivascular space compared to control. Since αvβ3 neutralization decreases but does not abrogate adherence of activated CD4 T lymphocytes to EGFL7, additional partners such as Notch^19,39^ could be implicated in this interaction, and would warrant future dedicated studies.

Based on our data, we conclude that EGFL7 can exert its beneficial impact in neuroinflammation by indirectly restricting leukocyte trafficking from the peripheral blood compartment to the perivascular space by promoting BBB quiescence (lower MCAM expression) and integrity, and limiting immune cell infiltration by acting as a brake to CD4 T lymphocytes trafficking from the perivascular space to the CNS parenchyma.

Together, we identified a beneficial role of EGFL7 in neuroinflammation, namely limiting CNS infiltration of pro-inflammatory immune cells, opening up a new perspective in MS therapeutics based on the promotion of BBB integrity and quiescence instead of immunosuppression or complete restriction of immune cell transmigration. In light of our findings, smaller EGFL7 agonists, in development for other diseases, could therefore constitute an appealing therapeutic avenue for MS. On the other hand, with the onset of clinical trials for anti-EGFL7 antibodies in oncology^10^, our results raise concerns over the risk of developing BBB dysfunction as a consequence of this treatment, and suggest that BBB integrity should be regularly monitored to timely detect potential CNS adverse effects.

## Methods

### Primary cultures of BBB-ECs

Human adult CNS tissue was obtained from patients undergoing epilepsy surgery (resection path). Informed consent and ethical approval was collected prior to surgery (Centre de Recherche du Centre Hospitalier de l’Université de Montréal/CRCHUM research ethic committee approval number HD04.046). Primary BBB-EC culture was established as previously described^40^. In brief, meninges were removed and CNS material was minced and resuspended in PBS. CNS material was washed to remove blood, then homogenized using loose-fitting Dounce homogenizer, and filtered on a 350 μm pore size mesh. Filtrate was subsequently passed twice through a 112 μm pore size mesh, collected, and treated with collagenase type IV (2 mg/ml) for 15 min at 37 °C. After inactivation with serum, filtrate was spun down and plated on gelatin-coated plates in EC culture media.

### Human immune cell isolation and culture

Informed consent was obtained from every donor prior to sample collection (CRCHUM ethic committee approval numbers SL05.022, SL05.023 and BH07.001; University Medical Center of the Johannes Gutenberg University Mainz ethic committee approval number 837.019.10(7028)). Peripheral blood was obtained by venous puncture from untreated RRMS subjects (neither disease-modifying therapy nor corticosteroids in the previous 3 months) and treated RRMS subjects (glatiramer acetate, β-interferons, teriflunomide or corticosteroids), as well as from healthy controls; immune cells were isolated as previously published^20^.

### Purification of EGFL7

Recombinant human (rh) and murine (rm) EGFL7 was purified from Sf9 insect cells by a baculovirus system. After infection, cells were lysed and loaded on 5 ml HisTrap FF columns. Flow-through was collected and fractions containing EGFL7 were purified by gel filtration on a Sephadex G75 column using an Äkta purifier chromatography system (GE Healthcare, Munich, Germany)^8^.

### Cytokine and CAM expression of memory CD4 T lymphocytes

To assess the effect of EGFL7 on expression of cytokines and CAMs, 6-well plates were coated overnight at 4 °C with either EGFL7 10 µg/ml or PBS. 0.5 million cells/well were seeded in 2 ml X-Vivo 15 medium. For activation, Dynabeads® Human T-Activator CD3/CD28 were added (12.5 µl/well) in combination with recombinant interleukin (IL)-2 (40 U/well). After 6 day of culture, cells were harvested, washed with FACS buffer, stained with anti-CD4-V450 (1:50), anti-CD4-PerCP (1:10), anti-CD8-AF700 (1:50), anti-CD106-APC (1:20), anti-CD146-AF647 (1:20), anti-CD196-PE (1:50), anti-CD11a-FITC (1:20), anti-IL17-AF647 (1:50), anti-IFN-V450-Horizon (1:50), anti-IL22-PECy7 (1:50), anti-IL4-FITC (1:20), anti-GMCSF-PE (1:50) fluorescent antibody and measured on a FACSCanto II (all antibodies from BD Bioscience). Surface expression of αvβ3 integrin by CD4+ T lymphocytes was measured using anti-αvβ3-APC (R&D) ex vivo or following activation in the presence of autologous monocytes as previously published^20^.

For lymphocyte intracellular cytokine staining, cells were activated for 4.5 h with 1 µg/ml ionomycin and 20 ng/ml phorbol 12-myristate 13-acetate (PMA) in the presence of 2 µg/ml brefeldin A. Cells were stained for surface antigens and then fixed and permeabilized in 4% paraformaldehyde. The samples were washed with Saponin Buffer and stained at 4 °C for 20 min. Samples were measured with a FACSCanto II and analysis was performed with FlowJo Software.

### Murine and human adhesion assay

CD4+ cells were magnetically isolated (positive selection) before activation with plate-bound anti-CD3 (2.5 µg/ml) and anti-CD28 (2 µg/ml) for 2–3 days. 96-well plates were coated overnight at 4 °C with EGFL7, ICAM-1, fibronectin (all 10 µg/ml), BSA 1% or PBS (total amount 100 µl/well). Activated CD4+ cells were harvested and CFSE-labeling was performed. Coating of the 96-well plate was removed and 100 µl of culture medium was immediately added. 100,000 activated CD4+ cells/well were added in 100 µl culture medium and the cells were left to adhere overnight. The wells were then washed twice with 200 µl PBS; 100 µl PBS was added to avoid drying of the adherent cells. The amount of adherent cells was measured by FITC signal detected with a fluorescence plate reader. When indicated, CFSE-labeled CD4+ cells were pre-treated for 1 h at 37 °C with either rm or rhEGFL7 10 µg/ml, αvβ3-inhibitor (murine: RGD-peptide 5.8 µg/ml; human: anti-αvβ3 neutralizing antibody 10 µg/ml), or appropriate control treatment (murine: RAD-peptide 5.9 µg/ml; human: rat serum 10% or mouse IgG1 10 µg/ml). Cells were then washed and added to coated wells.

### Immunostaining of CNS tissue

All human CNS material was processed and stored at the CRCHUM/Montreal. Frozen brain sections (hemispheric frontal, temporal, and parietal parenchyma) were prepared following standardized procedures. Brain sections from control subjects and different types of lesions/tissue from the same MS subject were processed and analyzed concurrently. Frozen sections of CNS autopsy specimens obtained from five RRMS patients (brain, *n* = 5 RRMS subjects, 5–7 sections/patient) and controls (brain, *n* = 5 control subjects, 5 sections/subject), and of CNS specimens obtained from mice following rapid intracardiac perfusion (brain, cerebellum, brainstem; sagittal sections) and spinal cord (lumbosacral, thoracic, and cervical; axial sections), *n* = 4–5 mice/group, 10 sections/mouse, were fixed in acetone and then transferred to ethanol, hydrated in PBS and blocked with 10% species-specific serum for 30 min at room temperature (RT). Sections were then incubated at RT for 60 min with primary antibodies diluted in 3% species-specific serum. Sections were washed with PBS and 0.05% Tween20 before secondary antibodies were incubated for 60 min at RT. Sections were mounted using either Mowiol mounting medium containing TO-PRO®3 (1:300); or incubated with 4′,6-diamidino-2-phenylindole (DAPI) 1:50,000 for 30 min before mounting with Prolong® Gold.

The following primary antibodies were used: mouse anti-human collagen IV (Abcam; 1:200) or goat anti-human collagen IV (Millipore; 1:80), mouse anti-human CD31 (BD Biosciences; 1:200), rabbit anti-human EGFL7 (clone 57; 1:100), mouse anti-human CD4 (BD Biosciences; 1:80), rat anti-mouse CD31 (BD Biosciences; 1:500), rat anti-mouse laminin-alpha4 (RnD systems; 1:500), donkey anti-mouse IgG-AF488 (Invitrogen; 1:500), rabbit anti-mouse collagen IV (BioRad; 1:100), rat anti-mouse CD4 (BD Biosciences AF647-labeled; 1:200), rabbit anti-mouse fibrinogen (Innovative Research; 1:1500), rabbit anti-mouse CD146 (Abcam; 1:200), rat anti-mouse CD106 (eBioscience; 1:100) and rat anti-mouse CD54 (eBioscience; 1:100). Fluorescence was visualized and acquired in a Leica Confocal Microscope secondary progressive platform. Image processing and analysis were performed using LAS and ImageJ software. Semi-quantitative analysis of EGFL7 expression in human CNS was reported as mean pixel intensity, as relative pixel intensity (mean pixel intensity × area) and as area normalized to total vessel area (EGFL7+ area/collagen IV+ area), using ImageJ software. Plasma protein leakage was determined by multiplying fluorescence intensity (mean pixels) and the area of leakage (number of pixels) of anti-mouse IgG-AF488^41^, using ImageJ software.

### Expression of CAMs by human BBB-ECs

BBB-ECs were grown to confluence on gelatin-coated 6-well plates (72 h) before inflammation with TNF/IFN-γ (100 U/ml) in the presence of rhEGFL7 (10 μg/ml) or vehicle. After 18 h, cells were harvested by scraping and quantitative PCR was performed as previously published^20^. Relative gene expression levels were determined using primers and TaqMan® FAM^TM^-labeled MGB probe for MCAM, VCAM-1, ICAM-1, and ALCAM and ribosomal 18 S (VIC®-labeled probe; Applied Biosystems). Gene-specific messenger RNA was normalized as compared to endogenous control 18 S.

### BBB transmigration assay

A modified Boyden Chamber assay was used to mimic the human BBB, as previously published^20^. BBB-ECs (3 × 10^4^ cells) were grown on gelatin-coated 3 µm pore size Boyden chambers in culture medium supplemented with 40% ACM for 72 h (to confluence). Inflamed condition consisted of 24 h exposure of BBB-ECs to TNF/IFN-γ (100 U/ml), followed by two washes with medium before performing the transmigration assay. When indicated, BBB-ECs were treated with rhEGFL7 (10 μg/ml) or vehicle for the last 24 h, and again 1 h before a suspension of 1 × 10^6^ T lymphocytes was added to the upper chamber and allowed to migrate for 18 h. All conditions were performed in triplicate. Absolute number of cells that transmigrated to the lower chamber was then assessed.

### In vivo BBB permeability

Permeability of the BBB was determined by intravenous injection of Cascade Blue labeled dextran (3 kDa) and dextran-TRITC (20 kDa); each in a concentration of 1 mg dissolved in 0.9% NaCl, as previously published^42^. Fifteen minutes after injection of the dextrans, 200 μl of blood was obtained by intracardiac puncture before immediate perfusion with ice-cold saline. CNS (whole brain and spinal cord) was removed and then placed in 1 ml of cold PBS and protected from light. The CNS samples were homogenized using progressively smaller needles and weighed afterwards. Blood and CNS proteins were precipitated in a next step by adding 1 ml of 60% trichloroacetic acid to the homogenized CNS samples. After removal of precipitates by centrifugation, the fluorescence was measured with a plate reader in a dark wall, clear bottom 96-well plate. The amount of fluorescent dye in the CNS tissue is depicted as a percentage of the fluorescence intensity found in the blood from the same mouse, normalized based on CNS weight. Immunoblotting for serum albumin (goat anti mouse serum albumin, abcam) was performed as previously published on whole brain and spinal cord^19^.

### Active EAE disease induction and scoring

For active EAE, typically 8 to 12-week-old C57Bl/6 mice, EGFL7-KO mice, microRNA miR-126/miR-126*-KO, *Egfl7fl/fl;Cdh5(PAC)-CreERT2* and respective WT or heterozygous (Het) littermates, and *Rag2*^*−/−*^*cgn*^*−/−*^ mice were immunized subcutaneously with 200 µg of myelin oligodendrocyte glycoprotein (MOG_35–55_) peptide, emulsified in complete Freund’s adjuvant, and supplemented with heat-inactivated M. tuberculosis (Hooke kit) according to the manufacturer’s instructions. Female mice were used for all experiments except for those with *Egfl7fl/fl;Cdh5(PAC)-CreERT2* and associated controls which used all males. Pertussis toxin 400 ng (Hooke kit) was administered intraperitoneally (i.p.) at the time of immunization and after 24 h. Before MOG_35–55_ immunization of male blood vessel-specific EGFL7-KOs (*Egfl7fl/fl;Cdh5(PAC)-CreERT2*) or respective controls, two doses of tamoxifen were given on day 3 and day 4 before EAE induction. To observe the effect of EGFL7 in EAE, rmEGFL7 or vehicle was administered i.p. in C57Bl/6 mice every other day, starting on day 4 after immunization. To test whether the observed effect of EGFL7-KO in EAE is mediated by the loss of EGFL7 or miR-126/miR-126* in immune cells, we performed active EAE in *Rag2*^*−/−*^*cgn*^*−/−*^ mice 4 weeks after reconstitution with 4 million CD4+ T lymphocytes from either EGFL7-KO or miR-126/miR-126*-KO versus their respective WT or Het littermates. Mice were evaluated for clinical symptoms on a daily basis using the following disease scoring system^43^: 0 = normal, 0.5 = tail weakness, 1 = tail paralysis, 1.5 = slow righting reflex, 2 = hind limb paresis, 2.5 = paralysis of one hind limb, 3 = bilateral hind limb paralysis, 3.5 = bilateral hind limb paralysis and fore limb paresis, 4 = paralysis of both hind limbs and one fore limb, 4.5 total paralysis of hind limbs and fore limbs (moribund), 5 = death (mice with a score above 3.5 were euthanized). The scoring was performed by an investigator blinded to the treatment group. Murine immune cell isolation from the spleen and whole CNS (whole brain and spinal cord) was performed at indicated time points during EAE following rapid intracardiac perfusion before processing for FACS analysis, as previously described^20,44^. All animal procedures were performed in accordance with the European Union normative for care and use of experimental animals, conducted according to the German Animal Protection Law and approved by the appropriate state committees for animal welfare.

### Expression of cytokine and CAM by murine CD4+ T lymphocytes

Murine immune cell isolation was performed as previously described^20,44^. To assess the effect of EGFL7 on the expression of cytokines and CAMs, after 5 days of activation with anti-CD3 and anti-CD28 (2 µg/ml), cells were seeded on 24-well plates which were coated overnight at 4 °C with either rmEGFL7 10 µg/ml or PBS. Cells were seeded in a concentration of 1 million cells/well in 2 ml mouse medium as previously published^45^. After 24 h, cells were harvested, washed and stained with anti-CD4-V450 (BD Biosciences; 1:100), anti-CD106-PeCy7 (BioLegend; 1:200), anti-CD146-PE (BioLegend; 1:100), anti-CD61-FITC (BD Biosciences; 1:100), anti-CCR6-AF647 (BD Biosciences; 1:100), anti-CD44-AF700 (BD Biosciences; 1:200), anti-CD25-PECy7 (BD Biosciences; 1:100), anti-CD49d-FITC (eBioscience; 1:50), anti-CD11a-PE (BD Biosciences; 1:200), anti-CD54-APC (BioLegend; 1:200). Surface expression of αvβ3 integrin by CD4 T lymphocytes was measured using anti-αvβ3-PE (SantaCruz; 1:200) ex vivo or following activation in the presence of antigen-presenting cells (APC) and anti-CD3.

For lymphocyte intracellular cytokine staining, cells were activated and processed for intracellular staining as described above. FoxP3 staining kit was used when appropriate. The following antibodies were used: anti-FoxP3-PECy7 (eBioscience; 1:50), anti-IL-4-PE (BD Biosciences; 1:50), anti-IL-10-APC (BD Biosciences; 1:200), anti-IL-17-FITC (eBioscience; 1:200), anti-TNFα-AF700 (BD Biosciences; 1:200) or anti-IFNγ-V450 (BD Biosciences; 1:200).

### Mouse models

Constitutive *egfl7*^*−/−*^ (EGFL7-KO) mice were generated and kindly provided by Weilan Ye (Genentech, USA)^15^. The KO was achieved by insertion of a retroviral gene trap vector upstream of intron 2 of the *egfl7* gene. The insertion leads to silencing of endogenous *egfl7* transcription; transcripts initiated from the inserted vector contain stop codons in all three frames, thus abolishing EGFL7 protein synthesis. Constitutive miR-126/miR-126*^−/−^ (miR-126/miR-126*-KO) mice were provided by Marc Tjwa (Goethe University, School of Medicine, Frankfurt am Main, Germany). The KO was achieved by removal of intron 7 of the *egfl7* gene that harbors *miR-126* and miR-126* genes as previously described^46^. The *Egfl7fl/fl;Cdh5(PAC)-CreERT2* is a tamoxifen-inducible KO of *egfl7* restricted to blood vessels, which preserves expression of miR-126/miR-126*^19^. The successful deletion of *egfl7* in Cadherin-5 (Cdh5) positive vessels after tamoxifen induced Cre-recombination is visualized by eGFP-signal.

### Fluorescence in situ hybridization and immunohistochemistry

Mice were sacrificed by cardiovascular perfusion with PBS. Brain and spinal cord were removed and embedded in Tissue-Tek O.C.T. compound, frozen on dry-ice and stored at −80 °C. Ten micrometer cryo sections were prepared, dried for 30 min at 37 °C and stored at −20 °C until further processing. Fluorescence in situ hybridization (FISH) was performed using QuantiGene ViewRNA ISH Cell Assay (Affymetrix). Expression of EGFL7 by CNS (cerebellum, brainstem, and spinal cord) endothelium in mice was visualized by a combination of FISH, using EGFL7-specific probes (mRNA), and immunofluorescence (IF) to identify claudin 5 (clone 4C3C2). Subsequent to a PBS wash, sections were incubated with blocking solution (10% goat serum plus 0.01% Triton X-100) at RT for 1 h. Sections were incubated with primary antibodies in blocking solution at 4 °C overnight. Subsequent to thorough washing, specific staining of antibody was revealed using species-specific fluorophore-conjugated secondary antibodies at RT for 1 h under light protection. Sections were washed in PBS and counterstained using DAPI 10 µg/ml at RT for 10 min. Sections were washed with distilled water and mounted in Aqua Poly Mount. Images were captured using a confocal microscope.

### Statistical analysis

Statistical analysis was performed using Prism (GraphPad software). Results are presented as the mean ± standard error of the mean (SEM), if not indicated otherwise. After assessing for distribution and when appropriate, paired or unpaired Student *t*-test, Mann-Whitney *U*-test, or one-way ANOVA, was performed. When indicated, two-way repeated measures ANOVA followed by a Bonferroni post hoc test was performed. Survival curve statistical analysis was performed with Mantel-Cox and Gehan-Breslow-Wilcoxon tests. *P* values < 0.05 were considered statistically significant. No randomization was applied.

### Data availability

Data that support the findings of this study are available from the corresponding author upon request.

## Electronic supplementary material

Supplementary Information
