# Supplementary material for: EGFL7 reduces CNS inflammation in mouse
Source: Nat Commun. 2018 Feb 26;9:819. doi: 10.1038/s41467-018-03186-z (PMC5827652; doi:10.1038/s41467-018-03186-z)
Supplement: Supplementary file 1 — Supplementary Information [file 41467_2018_3186_MOESM1_ESM.pdf]

Supplementary Information

EGFL7 reduces CNS inflammation in mouse

Larochelle and Uphaus et al.

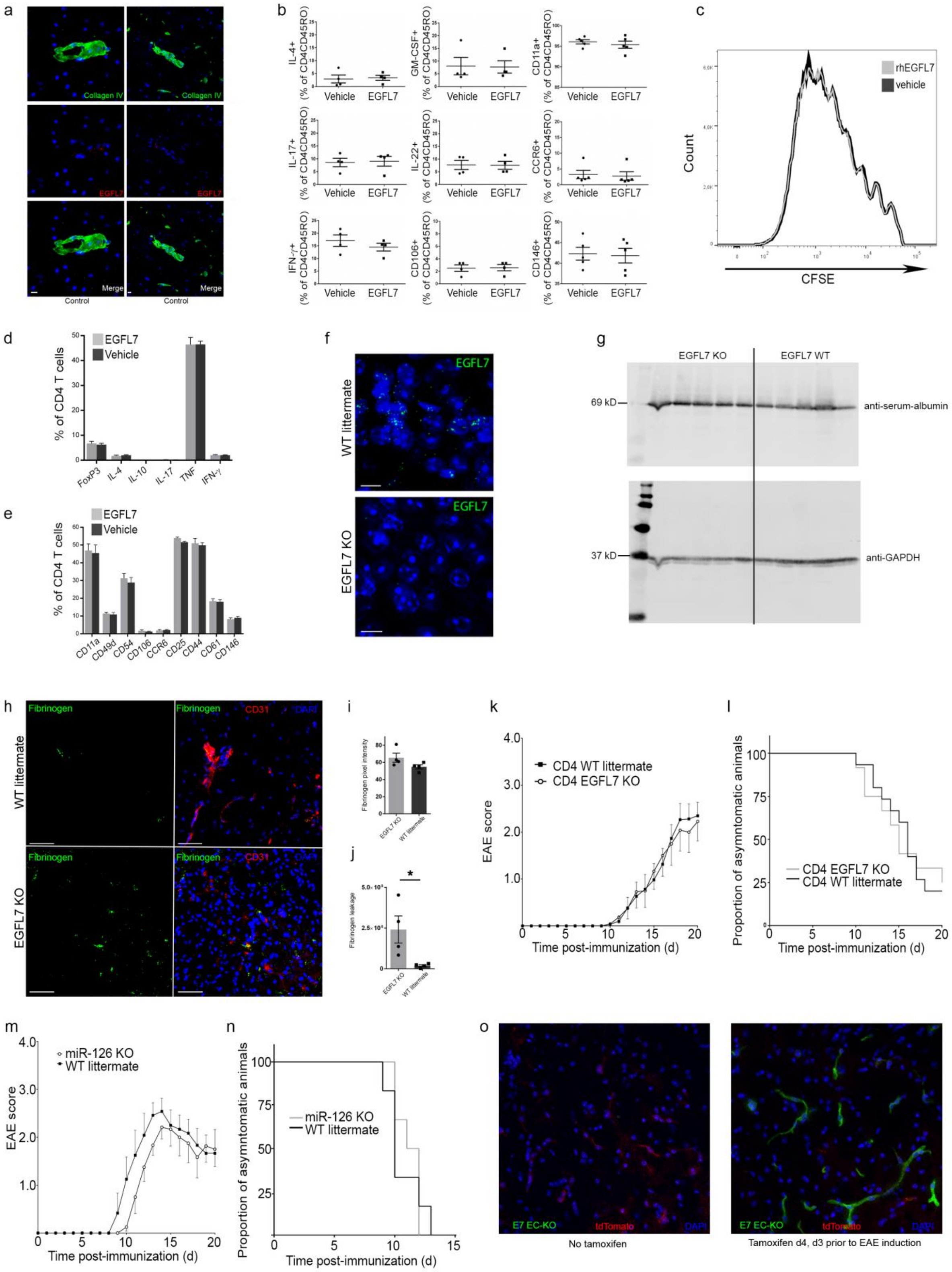

Supplementary Figure 1: The beneficial role of EGFL7 in neuroinflammation is not dependent on expression by CD4 T lymphocytes or expression of miR-126/miR-126*

a) Expression of EGFL7 (red) by CNS brain vessels (collagen IV, green) in human healthy controls (additional examples). Nuclei (DAPI) = blue. Scale bar = 10 µm. b) Expression of cytokines and cell adhesion molecules by human memory CD4 T lymphocytes activated for 6 d over cell culture plates pre-coated with rhEGFL7 or vehicle, as measured by flow cytometry. 1 dot = 1 donor. No significant difference was observed following analysis by paired Student's t-test. (c) Flow cytometry histogram showing proliferation of human memory CD4 T lymphocytes described in (b). Representative of n = 4 donors. (d) Expression of cytokines, FoxP3, and (e) activation markers and cell adhesion molecules by activated murine CD4 T lymphocytes put on cell culture plates pre-coated with rmEGFL7 or vehicle for 24 h before analysis by flow cytometry. n = 6 mice. No significant difference was observed following analysis by paired Student's t-test. (f) Representative EGFL7 fluorescence in situ hybridization (FISH; green) in EGFL7 knockout (KO) mice and wild-type (WT) littermates. Nuclei (DAPI) = blue. Scale bar = 10 µm. (g) Uncropped Western Blot images of figure 3h. (h-j) BBB integrity as measured by fibrinogen leakage was assessed by immunofluorescence on frozen sections (cerebellum, brainstem, spinal cord) from representative EGFL7-KO mice (lower panels) or WT littermates (upper panels). (h) Representative images from the spinal cord. Scale bar = 50 µm, EAE d10. (i, j) Semi-quantitative analysis of fibrinogen signal (green) according to (i) mean pixel intensity and (j) leakage of fibrinogen according to relative pixel intensity (mean pixel intensity X area). n ≥ 5 sections/group, from n = 4 mice/group, EAE d10. * p <0.05 (Mann-Whitney *U*-test). (k) EAE course and (l) proportion of asymptomatic animals over time in MOG_{35-55}-immunized *Rag2*$^{-/-}$*cgn*$^{-/-}$ mice reconstituted with CD4 T lymphocytes from EGFL7-KO mice (n = 11) versus from WT littermates (n = 13). (m) EAE course and (n) proportion of asymptomatic animals over time in MOG_{35-55}-immunized *miR-126/miR-126**-KO mice (open circles) versus WT littermates (black squares); n = 6-7 mice/group. No significant difference observed following analysis by two-way ANOVA for repeated measures and Mann-Whitney *U*-test (k, m). No significant difference following analysis by Mantel-Cox and Gehan-Breslow-Wilcoxon tests (l, n). (o) *Egfl7fl/fl;Cdh5(PAC)-CreERT2* male mice were treated either with tamoxifen (right panel) or vehicle (left panel) on d4 and d3 prior to EAE-induction. Successful deletion of EGFL7 by tamoxifen-inducible Cre-recombination is visualized by eGFP-signal (green).

Supplementary Table 1: EGFL7 knockout (KO) is associated with an earlier onset of EAE pathology compared to wild type (WT) littermates in two independent experiments

	Proportion of mice showing EAE clinical signs			
	EAE 1		EAE 2	
Day after immunization	EGFL7 KO	WT littermates	EGFL7 KO	WT littermates
5	0/5	0/5	0/7	0/7
6	0/5	0/5	0/7	0/7
7	0/5	0/5	1/7	0/7
8	2/5	0/5	2/7	0/7
9	2/5	1/5	2/7	0/7
10	5/5	1/5	3/7	0/7
11	5/5	4/5	6/7	0/7
12	5/5	4/5	6/7	1/7
